# Association of antinuclear antibodies with the risk of intracranial arterial stenosis

**DOI:** 10.18632/aging.102685

**Published:** 2020-01-21

**Authors:** Yong-An Sun, Qiu Han, Xiao-He Hou, Xian-Zhen Peng, Lin Tong, Xu Zheng, Jin-Tai Yu, Lan Tan

**Affiliations:** 1 Department of Neurology, First Affiliated Hospital of Kangda College, Nanjing Medical University, Lianyungang, China; 2 Department of Neurology, Qingdao Municipal Hospital, Qingdao Clinical Medical College, Nanjing Medical University, Qingdao, China; 3 Department of Neurology, Huai’an First People’s Hospital, The Affiliated Huai’an No.1 People’s Hospital of Nanjing Medical University, Huai’an, China; 4 Department of Neurology, Qingdao Municipal Hospital, Qingdao University, Qingdao, China; 5 Department of Public Health and Preventive Medicine, Kangda College of Nanjing Medical University, Lianyungang, China; 6 Department of Neurology, Yantai Affiliated Hospital of Binzhou Medical Universtiy, Yantai, China; 7 Department of Clinical Laboratory, Qingdao Municipal Hospital, Qingdao University, Qingdao, China; 8 Department of Neurology and Institute of Neurology, Huashan Hospital, Shanghai Medical College, Fudan University, Shanghai, China

**Keywords:** intracranial arterial stenosis, antinuclear antibodies, magnetic resonance angiography, acute ischemic cerebrovascular disease

## Abstract

The prevalence of intracranial arterial stenosis (IAS) as well as antinuclear antibody (ANA) positivity was found to be higher in Asians than that in the Western population. To investigate the relation of ANAs with IAS in patients with acute ischemic cerebrovascular disease, we enrolled 2492 patients with acute ischemic stroke or transient ischemic attack into the study. All the patients were categorized into 3 groups according to the IAS burden. Multinomial logistic regression analyses were used in statistical analysis. The positive rate of ANAs in the IAS ≥ 2 group was higher than that in the single IAS group and the no IAS group (p<0.001). The adjusted odds ratio (OR) for IAS ≥ 2 in ANAs-positive patients was 3.737 (95%CI=2.676-5.220, p<0.001) compared with the ANAs-negative patients. ANAs were associated with multiple IAS rather than single IAS in both male and female subgroups. Besides, ANAs were significantly associated with single and multiple IAS in individuals ≤ 60 years. However, ANAs were only associated with two or more IAS in two age groups (between 61 to 75 years and >75 years old). In summary, ANAs are associated with IAS in patients with acute ischemic cerebrovascular disease.

## INTRODUCTION

Intracranial arterial stenosis (IAS) is one of the most common causes of ischemic stroke. IAS is more prevalent among Asians than in Whites [1, 2]. Among the causes of IAS, arteriosclerotic stenosis accounts for the vast majority [3]. In addition, arteritis, arterial dissection and moyamoya disease are also the causes of IAS [4, 5]. Antinuclear antibodies (ANAs) are a series of autoantibodies targeting various nuclear and cytoplasmic components of cells. Studies have found that there are differences in the positive rate of ANAs among different races, and healthy non-Caucasians and African Americans present a higher prevalence of ANAs compared with whites [6, 7]. It has been reported that the positive ANAs can accompany large vessel vasculitis [8]. Other studies showed that the positive ANAs are associated with increased risk of early atherosclerosis in Sjögren’s syndrome (SS) patients [9]; there is a close relationship between serum anti SSB antibody and vascular endothelial injury in SLE patients [10]; high titers of serum ANAs are associated with the presence of coronary atherosclerosis [11]. The preliminary study suggests that positive ANAs may be a risk factor for cerebral infarction [12]. However, the association of ANAs with intracranial atherosclerosis or stenosis remains unclear. The high prevalence of IAS and ANA positivity in Asians aroused our interest in exploring the relationship between them. Is there some relationship between high incidences of IAS and ANA positivity in Asians? Therefore, we explored the association between ANAs and IAS in patients with acute ischemic cerebrovascular disease.

## RESULTS

### Patient characteristics

The baseline demographic and clinical characteristics, laboratory findings are shown in Table 1. A total of 2,492 patients were included in our statistical analysis. There were 1056 (42.4%) males and 1436 (57.6%) females with a median age of 70 (63-78) years. The positive rates of ANAs were significantly different among the multiple IAS group, single IAS group and no IAS group (p<0.001).

**Table 1:** Demographic and clinical characteristics of the study participants.

**Characteristics**	**IAS burden=0 (n=1371)**	**IAS burden=1 (n=637)**	**IAS burden≥2 (n=484)**	**z /χ^2^**	**P**
Age, years (Mean ± SD)	67.14±11.20	71.02±10.01	73.91±10.13	154.796	<0.001
Female, n (%)	887(64.70)	335(52.59)	214(44.21)	70.339	<0.001
MAP, mm Hg, median (IQR)	100(92-107)	100(93-107)	101(93-109)	11.212	0.004
History of stroke, n (%)	172(12.55)	138(21.66)	169(34.92)	118.595	<0.001
Hypertension, n (%)	929(67.76)	502(78.81)	407(84.09)	60.561	<0.001
Diabetes, n (%)	392(28.59)	238(37.36)	252(52.07)	87.652	<0.001
Dyslipidemia, n (%)	1052(76.73)	483(75.82)	370(76.45)	0.199	0.905
Coronary heart disease, n (%)	555(40.48)	313(49.14)	248(51.24)	23.301	<0.001
Atrial fibrillation, n (%)	51(3.72)	58(9.11)	77(15.91)	80.288	<0.001
Hyperuricemia, n (%)	530(38.66)	274(43.01)	222(45.87)	8.877	0.012
Smoking habits, n (%)	258(18.82)	157(24.65)	175(36.19)	59.958	<0.001
Drinking habits, n (%)	180(13.13)	105(16.48)	127(26.24)	44.559	<0.001
FBG, mmol/l, median (IQR)	5.25(4.73-6.03)	5.23(4.69-6.36)	5.57(4.82-7.72)	29.822	<0.001
SUA, umol/l, median (IQR)	334.33(281.55-384.44)	332.48(277.39-406.16)	347.54(286.50-419.87)	8.995	0.011
Homocysteine, umol/l, median (IQR)	6.9(5.8-8.2)	7.1(5.9-8.5)	7.6(6.5-9.6)	39.067	<0.001
Lipoprotein(a), mg/dl median (IQR)	17.26(8.79-34.35)	18.22(7.61-37.65)	22.45(10.92-42.75)	15.250	<0.001
Triglycerides, mmol/l, median (IQR)	1.30(0.97-1.79)	1.22(0.93-1.59)	1.24(0.91-1.69)	15.180	<0.001
Total cholesterol, mmol/l, median (IQR)	4.94(4.23-5.72)	4.88(3.96-5.77)	4.51(3.77-5.47)	33.037	<0.001
HDL, mmol/l, median (IQR)	1.15(0.97-1.38)	1.14(0.95-1.31)	1.02(0.89-1.23)	59.970	<0.001
LDL, mmol/l, median (IQR)	3.03(2.50-3.60)	3.08(2.40-3.67)	2.77(2.27-3.43)	25.359	<0.001
Neutrophil, ×10^9^/l, median (IQR)	3.32 (2.60-4.30)	3.53(2.70-4.65)	3.81(2.88-5.00)	31.961	<0.001
Lymphocyte, ×10^9^/l, median (IQR)	1.99(1.59-2.45)	1.90(1.42-2.39)	1.86(1.48-2.30)	22.238	<0.001
Autoimmune disease, n (%)	72(5.25)	30(4.71)	26(5.37)	0.331	0.848
ANAs(positive), n (%)	243(17.72)	145(22.76)	184(38.02)	83.309	<0.001

IAS, Intracranial arterial stenosis; MAP, mean arterial pressure; SBP, systolic blood pressure; DBP, diastolic blood pressure; MAP=(SBP+2×DBP)/3; FBG, fasting blood glucose; SUA, serum uric acid; HDL, high density lipoprotein; LDL, low density lipoprotein; ANAs, antinuclear antibodies. IAS burden was defined as the total number of intracranial arteries with significant stenosis (≥50% stenosis).

### Association between ANAs and IAS burden

Association between ANAs and IAS burden is shown in Table 2. Multivariate logistic regression analysis of the overall subjects showed that although ANAs was associated with the single IAS (Adjusted OR1=1.451,95% CI=1.142-1.843, p=0.002), the association did not reach statistical significance after adjustment for all potential confounders (adjusted OR2=1.252, 95%CI=0.920-1.705, P=0.153). After adjusting for all confounding factors, compared with the ANAs-negative patients, the adjusted OR for two or more IAS in ANAs-positive patients was 3.737 (95% CI=2.676-5.220, p<0.001).

**Table 2:** Association between ANAs and IAS burden.

		**Single IAS vs No IAS**		**IAS ≥2 vs No IAS**	
		**OR (95% CI)**	**P value**	**OR (95% CI)**	**P value**
ANAs (+) vs ANAs (-)	Crude OR	1.368(1.086,1.724)	0.008	2.847(2.262,3.583)	<0.001
	Adjusted OR1	1.451(1.142,1.843)	0.002	3.468(2.677,4.494)	<0.001
	Adjusted OR2	1.252(0.920,1.705)	0.153	3.737(2.676,5.220)	<0.001

IAS, Intracranial arterial stenosis; ANAs, antinuclear antibodies; OR, odds ratio; CI, confidence interval; Adjusted OR1, adjusted for age, gender, MAP, stroke history, hypertension, diabetes mellitus, coronary heart disease, atrial fibrillation, hyperuricemia, smoking habits and drinking habits; Adjusted OR2, adjusted for the items in OR1 plus blood glucose, serum uric acid, serum homocysteine, serum triglyceride, serum cholesterol, serum high density lipoprotein, serum low density lipoprotein, neutrophil and lymphocyte counts.

### Subgroup analysis in different gender groups

Subgroup analysis in different gender groups showed that the risks for two or more IAS in ANAs-positive male and female subgroups were 3.142 (95%CI=1.886-5.234, P<0.001) and 4.525 (95%CI=2.841-7.207, P<0.001) times higher, respectively, compared with the ANAs-negative patients. However, among the male and female subgroups, there was no significant association between ANAs positivity and a single IAS (Table 3).

**Table 3:** Association between ANAs and IAS burden among different gender.

	**Gender**		**Single IAS vs No IAS**		**IAS ≥2 vs No IAS**	
			**OR (95% CI)**	**P value**	**OR (95% CI)**	**P value**
ANAs (+) vs ANAs (-)	Male (n=1056)	Crude OR	1.308(0.917,1.866)	0.139	2.250(1.597, 3.169)	<0.001
		Adjusted OR1	1.363 (0.939,1.980)	0.104	2.792 (1.895,4.114)	<0.001
		Adjusted OR2	1.051 (0.643,1.716)	0.843	3.142 (1.886,5.234)	<0.001
	Female (n=1436)	Crude OR	1.409(1.036, 1.918)	0.029	3.699(2.684,5.099)	<0.001
		Adjusted OR1	1.534(1.116,2.110)	0.008	4.294(3.015,6.115)	<0.001
		Adjusted OR2	1.375(0.905,2.090)	0.136	4.525(2.841,7.207)	<0.001

IAS, Intracranial arterial stenosis; ANAs, antinuclear antibodies; OR, odds ratio; CI, confidence interval; Adjusted OR1, adjusted for age, gender, MAP, stroke history, hypertension, diabetes mellitus, coronary heart disease, atrial fibrillation, hyperuricemia, smoking habits and drinking habits; Adjusted OR2, adjusted for the items in OR1 plus blood glucose, serum uric acid, serum homocysteine, serum triglyceride, serum cholesterol, serum high density lipoprotein, serum low density lipoprotein, neutrophil and lymphocyte counts.

### Subgroup analysis in different age groups

Subgroup analysis according to age showed that the associations between ANAs and IAS burden were not the same in different age groups. ANAs positivity was significantly associated with single and multiple IAS in individuals ≤ 60 years (adjusted OR2=3.855, 95% CI=1.625-9.144, p=0.002 for single IAS; adjusted OR2=5.655, 95%CI=1.574-20.324, p=0.008 for two or more IAS). However, ANAs positivity was only associated with two or more IAS in individuals aged between 61 to 75 years (adjusted OR2=4.393, 95% CI=2.674-7.216, p<0.001) and those older than 75 years old (adjusted OR2=4.083, 95% CI=2.245-7.423, p<0.001) (Table 4).

**Table 4:** Association between ANAs and IAS burden among different ages.

	**Age**		**Single IAS vs No IAS**		**IAS ≥2 vs No IAS**	
			**OR (95% CI)**	**P value**	**OR (95% CI)**	**P value**
ANAs (+) vs ANAs (-)	Age≤60 (n=485)	Crude OR	3.531(2.091, 5.961)	<0.001	3.852(1.967, 7.540)	<0.001
		Adjusted OR1	4.027 (2.308, 7.026)	<0.001	4.536(2.104,9.781)	<0.001
		Adjusted OR2	3.855 (1.625, 9.144)	0.002	5.655(1.574,20.324)	0.008
	60<Age≤75 (n=1173)	Crude OR	1.085(0.776,1.516)	0.634	3.074(2.187, 4.320)	<0.001
		Adjusted OR1	1.217(0.858,1.725)	0.271	4.163(2.822, 6.142)	<0.001
		Adjusted OR2	1.038(0.660,1.631)	0.872	4.393 (2.674,7.216)	<0.001
	Age>75 (n=834)	Crude OR	1.221(0.801,1.862)	0.354	2.491(1.699,3.651)	<0.001
		Adjusted OR1	1.238(0.802,1.911)	0.336	3.034 (2.004,4.592)	<0.001
		Adjusted OR2	0.831 (0.445,1.550)	0.560	4.083 (2.245, 7.423)	<0.001

IAS, Intracranial arterial stenosis; ANAs, antinuclear antibodies; OR, odds ratio; CI, confidence interval; Adjusted OR1, adjusted for age, gender, MAP, stroke history, hypertension, diabetes mellitus, coronary heart disease, atrial fibrillation, hyperuricemia, smoking habits and drinking habits; Adjusted OR2, adjusted for the items in OR1 plus blood glucose, serum uric acid, serum homocysteine, serum triglyceride, serum cholesterol, serum high density lipoprotein, serum low density lipoprotein, neutrophil and lymphocyte counts.

### Dose-effect relationship between ANAs and IAS burden

To further validate the association between ANAs and IAS, we analyzed the association between the number and grade of ANAs and IAS burden in the 572 ANAs-positive patients. The distribution and grades of positive ANAs are shown in Supplementary Figure 1. Defined ANAs-Count as the sum of ANAs positivity items in ANAs-positive individuals; defined ANAs-Max as the highest grade of ANAs positivity items in ANAs-positive individuals (+=1, ++=2, +++=3).

The IAS burden was taken as a dependent variable and the ANAs-Count as well as ANAs-Max were taken as independent variables. After adjusting for all confounding factors, multivariate logistic regression analysis showed that there was no significant association of ANAs-Count and ANAs-Max with single or multiple IAS in the 572 ANAs-positive patients (Table 5).

**Table 5:** Association between the number and grade of positive ANAs and IAS burden.

		**Single IAS vs No IAS**		**IAS ≥2 vs No IAS**	
		**OR (95% CI)**	**P value**	**OR (95% CI)**	**P value**
ANAs-Count	Crude OR	1.080 (0.709, 1.646)	0.719	1.091 (0.733, 1.625)	0.668
	Adjusted OR1	1.239 (0.807, 1.903)	0.327	1.159 (0.731, 1.839)	0.530
Adjusted OR2	1.099 (0.599, 2.019)	0.760	1.545 (0.892, 2.675)	0.121
ANAs -Max	Crude OR	0.978 (0.809, 1.183)	0.819	0.928 (0.778, 1.107)	0.406
	Adjusted OR1	0.989 (0.811, 1.205)	0.910	1.004(0.826, 1.221)	0.965
	Adjusted OR2	0.899 (0.688, 1.175)	0.436	0.897 (0.694, 1.160)	0.408

IAS, Intracranial arterial stenosis; ANAs, antinuclear antibodies; OR, odds ratio; CI, confidence interval; ANAs-Count: the number of positive antinuclear antibody items in ANAs positive individuals; ANAs-Max: the highest grade of positive ANAs in ANAs positive individuals (+=1, ++=2, +++=3); Adjusted OR1, adjusted for age, gender, MAP, stroke history, hypertension, diabetes mellitus, coronary heart disease, atrial fibrillation, hyperuricemia, smoking habits and drinking habits; Adjusted OR2, adjusted for the items in OR1 plus blood glucose, serum uric acid, serum homocysteine, serum triglyceride, serum cholesterol, serum high density lipoprotein, serum low density lipoprotein, neutrophil and lymphocyte counts. Dependent variable: the IAS burden (no IAS, single IAS, IAS≥2); Independent variables: the ANAs-Count and ANAs-Max; The reference category is ‘No IAS’.

## DISCUSSION

In the present study, we explored the relationship between ANAs and IAS. Our results showed that the positivity rates of ANAs among the three different IAS burden groups were significantly different. The positivity rate of ANAs in ≥2 IAS group was higher than those in single IAS group and no IAS group. Further analysis suggested that ANAs positivity had some association with multiple IAS in patients with acute ischemic cerebrovascular disease. The reason why ANAs are significantly associated with multiple IAS rather than single IAS is not clear. Studies have found that certain risk factors, such as an increased systolic blood pressure in the morning [13] and diabetes [14], were also associated with multiple intracranial stenoses rather than single stenosis in patients, suggesting the extensive effects of these virulence factors (including ANAs) on IAS. Since our study was based on cross-sectional data, the causal relationship between ANAs and IAS is not certain. In terms of the interaction mechanism between ANAs and IAS, there are several possible scenarios. Firstly, it is possible that some misregulation of immune and inflammatory responses independently leads to increased development of atherosclerosis and increased incidence of ANA positivity. Secondly, existing intracranial artery disease may lead to ANA seroconversion. Thirdly, cerebral infarction caused by vascular occlusion may also be sufficient to lead to leakage of intracellular antigens that can induce the production of ANAs. Fourthly, the presence of ANAs in the circulation may contribute to the pathogenesis of atherosclerosis [11]. Although there is no direct evidence for these four scenarios, the results of this study at least suggests that it merits further investigation. In this study, the causal relationship between multiple IAS and ANAs remain unclear. It is possible that severe enough angiopathy and antigen exposure can lead to ANA positive seroconversion, or positive ANAs have extensive damage to blood vessels, which needs to be explored in the longitudinal research in the future.

Other studies have found ANA-related cardiovascular risk may involve pathways distinct from traditional risk factors, including dysregulation of endothelial cells and the immune system [15]. It has been suggested that positive ANAs were part of the antiphospholipid antibody syndrome (APS), which can cause atherosclerosis, proliferation of smooth muscle cells and major vascular occlusion [12, 16]. The studies mentioned above suggested that ANAs may be directly involved in the pathogenesis of IAS in ANAs-positive patients. In addition, it is noteworthy that studies have found that ANAs not only damage vascular endothelium and lead to arteriosclerosis, but also promote the thrombotic tendency [17], which may be considered as the two pathological mechanisms of arterial stenosis and occlusion caused by ANAs.

Subgroup analysis according to gender showed that the results are similar to those in the general population. ANAs positivity was only associated with multiple IAS in both male and female subgroups, but was not significantly associated with single IAS. Subgroup analysis according to age showed that ANAs positivity was significantly associated with single and multiple IAS in the ≤ 60 years subgroup. However, ANAs positivity was only significantly associated with multiple IAS in the subgroup over 60 years old. Studies have found the attributable risk of hypertension, diabetes mellitus, and coronary artery disease for intracranial stenosis was lower among patients aged ≤45 years than that among patients aged >45 years [18]. Non-atherosclerotic disease is an important etiology of intracranial stenosis in young patients [19]. However, in older patients with intracranial stenosis, more metabolic factors such as hyperuricemia are involved [20]. Although the causal relationship between ANAs and IAS is not certain, we speculate that one of the possible reasons is that among all the patients in the ≤ 60 years subgroup as well as patients with multiple IAS in the group over 60 years old, the immune factors such as ANAs are involved in the occurrence of IAS. Among the patients with multiple IAS in the subgroup over 60 years old, there are more non-immunologic factors involved in the occurence of IAS. ANAs positivity in these patients may be just an epiphenomenon [21]. Our results indicate that ANAs have different associations with IAS in different age groups. The reason needs to be further investigated.

Our results did not show a significant association of the grades of ANAs titers or the count of ANAs items with the IAS burden in ANAs-positive patients. A study by Blann and his colleagues showed that although there was vascular endothelial damage in SS patients, no significant correlation was observed between ANA titers and serum levels of von Willebrand factor antigen which as an index of damage to the endothelium [21]. Although the validity of Willebrand factor antigen as an indicator of vascular endothelial damage is uncertain, we believe that the association between ANAs and IAS mainly depends on whether the ANAs are positive rather than ANAs titers and numbers of positive ANAs according to our results mentioned above.

Irrespective of the mechanisms which underlie the association, the strength of the association between ANA positivity and IAS suggests that the ANA positivity may be useful as an additional noninvasive diagnostic tool for identifying subjects at risk of IAS in the ischemic cerebrovascular disease patients. Our study is preliminary. If our results are replicated in larger cohorts, then ANA analysis may be a useful addition to the battery of tests presently used to diagnose the presence of IAS. ANA testing in acute ischemic cerebrovascular disease patients with IAS may also be helpful in exploring the pathogenesis of IAS.

There are several limitations in our study that should be mentioned. First, we cannot rule out the possibility that there were patients with congenital IAS and arterial dissection in our subjects. At the same time, the possibility that MRA has an exaggerated effect on the judgment of intracranial stenosis cannot be absolutely excluded. Second, we defined IAS burden as the number of intracranial arteries with significant stenosis, but it sometimes can't accurately reflect the overall severity of individual's intracranial lesions. Third, our study enrolled patients who were predisposed to IAS; therefore, the results should be interpreted with caution and cannot be generalized to other disease populations. Finally, we only analyzed the imaging stenosis for intracranial large arteries, but the pathological nature of stenotic lesions cannot be determined. In future studies, we will use high-resolution MR and vascular wall imaging techniques to further study the relationship between ANAs and IAS.

## CONCLUSIONS

In summary, our results indicate that ANAs are associated with an increased risk of IAS in patients with acute ischemic cerebrovascular disease. It’s also shown that ANAs have different effects on the increased risk of IAS in ischemic cerebrovascular disease patients with different ages and genders. The significance of ANAs for IAS mainly depends on whether the ANAs are positive rather than ANAs titers and numbers of positive ANAs. It may be of great significance to detect ANAs in patients with ischemic cerebrovascular disease who may have IAS.

## MATERIALS AND METHODS

### Study participants

Study subjects with acute ischemic stroke (AIS) (<7 days of onset) or transient ischemic attack (TIA) confirmed by neuroimaging were prospectively recruited from September 2014 to February 2018 in the department of neurology at Qingdao Municipal Hospital. The diagnosis of AIS and TIA conforms to the standards of the American Heart Association/American Stroke Association [22, 23]. We excluded individuals who:(1) less than 40 years old; (2) had undergone incomplete vascular imaging and laboratory tests; (3) had severe stenosis (≥70%) or occlusion confirmed by doppler ultrasonography of extracranial carotid artery, brachiocephalic trunk, subclavian artery, and extracranial vertebral artery; (4) had undergone intracranial or extracranial cerebral artery stenting or balloon angioplasty; (5) had undergone carotid endarterectomy; (6) were diagnosed with moyamoya disease; (7) had been clinically diagnosed with cerebral embolism; (8) did not provide written informed consent.

On admission, the information was collected and recorded in detail, such as baseline data, age, gender, blood pressure, and risk factors for ischemic stroke. In addition to routine blood tests and medical history collection, ANA test and MRA were performed in patients who agreed and signed an informed consent form as well. This study was approved by the Institutional Ethics Committee of Qingdao Municipal Hospital. Finally, 2492 patients were included and divided into three groups according to the vascular lesion burden showed by MRA.

### Ethics statement

This study was conducted in accordance with the Helsinki declaration and the protocol of this study was approved by the Institutional Ethics Committees of Qingdao Municipal Hospital. The written informed consent was obtained from all studied participants directly or from the authorized person.

### Definition of diseases and risk factors

Hypertension: defined as a history of hypertension or required regular treatment with antihypertensive agents before admission or diagnosed at discharge. Diabetes mellitus: defined as a history of diabetes mellitus or being treated for diabetes mellitus or glycosylated hemoglobin≥7% or diagnosed at discharge. Dyslipidemia: history of dyslipidemia, or currently taking lipid-lowering drugs for hyperlipidemia, or fit the following criterion: the serum total cholesterol (TC) >5.2mmol/L, or low-density lipoprotein (LDL) > 3.37mmol/L, or high-density lipoprotein (HDL) < 1.04 mmol/L, or triglyceride > 1.7mmol/L. Hyperuricemia was defined as serum uric level >357ummol/L or currently taking uric-acid-lowering drugs. History of previous stroke: once suffering from cerebral infarction or cerebral hemorrhage. Coronary heart disease: previous history of coronary heart disease or diagnosed at discharge. Atrial fibrillation: patients with atrial fibrillation diagnosed by electrocardiogram or previously diagnosed as paroxysmal atrial fibrillation. In addition, smoking habit was defined as a patient who had smoked continuously for 6 months ≥1 cigarette a day. Drinking habit was defined as the average alcohol consumption on average for more than half a year from past or present ≥2 units/d (16g/d).

### Detection of ANA and blood biochemical analysis

Three milliliters of fasting venous blood samples were drawn with a non-anticoagulant vacuum tube in the morning, and were preserved at -80°C until antinuclear antibody (ANA) and ANAs tests were done. As recommended by the manufacturer, all the blood samples were screened for ANA with indirect immunofluorescence technique (IFAT) on HEp-2 cells by using the commercially available kit ANA HEp-2 (HEp-2 cells, Euroimmun Hangzhou medical laboratory diagnostics GmbH, Hangzhou, China). ANA titers of at least 1:100 were considered positive. Simultaneously, blood samples were identified by immunoblotting for ANAs spectrum (anti-nRNP, Sm, SS-A, Ro-52, SS-B, Scl-70, PM-Scl, Jo-1, CENP-B, PCNA, dsDNA, Nukleosome, Histone, Ribosomal P protein, AMA-M2) using individual ENA kit (Euroimmun Hangzhou medical laboratory diagnostics GmbH, Hangzhou, China). According to the instructions, the procedures were carried out and the results were interpreted based on the degree of staining of the antigen colored ribbons. As recommended by the manufacturer, the results are assessed to be (-), (+), (++) (+++). Patients who are positive for the ANA screening test and/or are positive for one or more of the specific ANAs are considered to be ANAs-positive individuals. Blood biochemical analysis was conducted by BECKMAN COULTER (AU5800) automatic biochemistry analyzer (Beckman Coulter, Inc. Brea, USA).

### Imaging assessment

All the patients underwent conventional MRI on a 3.0-T MR scanner (Signa Excite HD 3.0T system, GE Medical Systems, USA) within two weeks following admission. MRI sequences included three-dimensional time of flight magnetic resonance angiography (3D TOF MRA), T2/ T1-weighted imaging, fluid-attenuated inversion recovery sequences (FLAIR), and diffusion-weighted imaging (DWI). MR angiograms were obtained using the following imaging parameters: repetition time/echo time (TR/TE), 21/3ms, flip angle 15°, the field of view (FOV) 240mm×240mm, matrix 320×160, and slice thickness 0.7mm without interslice gaps. All MRI/MRA images were stored in digital format and were read independently by two readers (Yongan Sun and Lin Tong) who were blinded to subjects’ clinical information. A third appointed senior reader would decide the final score if there were disagreements of larger than 10% on the degree of stenosis.

The following arterial segments were assessed: bilateral intracranial internal carotid arteries (ICA), middle cerebral arteries (MCA) M1/M2, anterior cerebral arteries (ACA) A1/A2, posterior cerebral arteries (PCA) P1/P2, intracranial vertebral arteries (V4) and basilar artery (BA). Based on the original and reconstructed images, the severity of intracranial stenosis on MRA was classified into four grades (no or <50% stenosis, 50% to 69% stenosis, 70% to 99% stenosis, and occlusion) using the published method Warfarin-Aspirin Symptomatic Intracranial Disease (WASID) study [24]. Focal flow void found on MRA with distal filling was regarded as severe stenosis (70%–99%). Absence of distal filling on MRA would be regarded as 100% occlusion. For the internal carotid artery, the intracranial location was defined as the distal end of the ophthalmic artery [25]. A significant artery stenosis lesion was defined as ≥50% stenosis or occlusion of the major intracranial cerebral arteries. IAS burden was defined as the total number of intracranial arteries with significant stenosis (≥50% stenosis) [26]. According to their IAS burden, the patients were divided into the group with no significant stenosis and the group with significant stenosis. The latter was further divided into single stenosis group and multiple stenosis group.

### Statistical analyses

Continuous variables that conform to a normal distribution, were expressed as mean ± standard deviation; continuous variables that do not conform to a normal distribution, were expressed as median and interquartile range (IQR). Continuous variables were compared by Wilcoxon test and categorical variables were compared by Chi-square test. In the regression analysis, if the parallel test hypothesis was rejected, a multinomial logistic regression model was used. In logistic regression model, OR1 was adjusted for stroke risk factors (age, gender, mean arterial pressure, stroke history, hypertension, diabetes mellitus, coronary heart disease, atrial fibrillation, hyperuricemia, smoking habits and drinking habits), and OR2 was adjusted for the items in OR1 plus blood laboratory results (blood glucose, serum uric acid, serum homocysteine, serum triglyceride, serum cholesterol, serum high density lipoprotein, serum low density lipoprotein, neutrophil and lymphocyte counts). All P values were 2-sided and evaluated at the 0.05 α level. All analyses were performed in Stata 12.0 (Stata Corp, College station, TX).

## Supplementary Material

Supplementary Figure 1
