# Supplementary material for: Association of antinuclear antibodies with the risk of intracranial arterial stenosis
Source: Aging (Albany NY). 2020 Jan 21;12(2):1322–31. doi: 10.18632/aging.102685 (PMC7053607; doi:10.18632/aging.102685)
Supplement: Supplementary Figure 1 [file aging-12-102685-s001..pdf]

SUPPLEMENTARY FIGURE

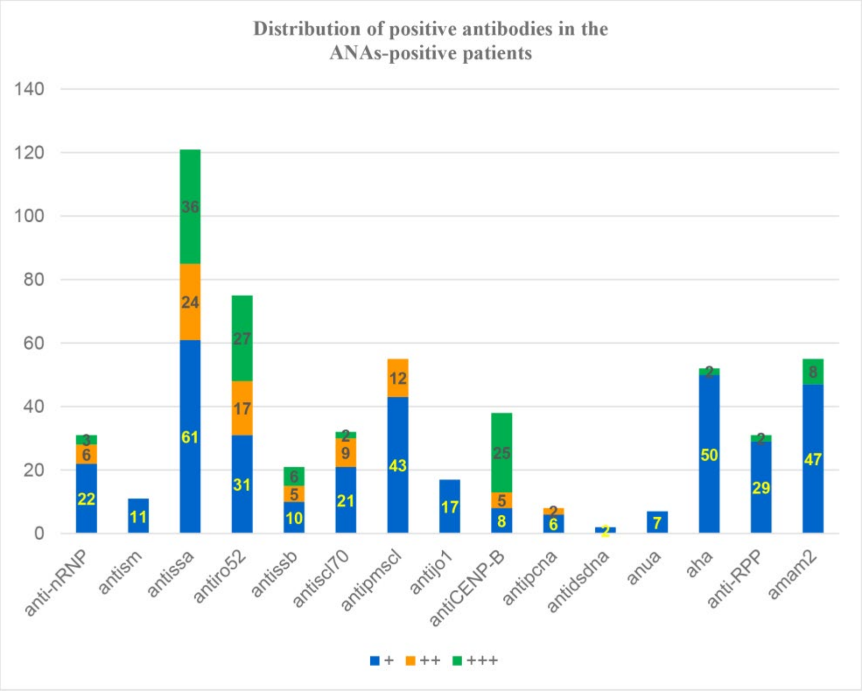

Supplementary Figure 1. Grades and distribution of positive antibodies in the ANAs-positive patients. Of the 15 antibodies in the ANAs-positive patients, The autoantibodies with the top-5 positive frequencies were anti-SSA 121 times (according to the staining intensity of the antigen band, + 61 cases, ++24 cases, +++36 cases) ,followed by anti-AMA-M2 positive 77 times (+ 47 cases, ++22 cases, +++8 cases); anti-Ro-52 positive 76 times (+ 31 cases, ++17 cases, +++27 cases); anti-AHA positive 75 times (+ 50 cases, ++23 cases, +++2 cases,) anti-PM/SCL positive 55 times(+ 43 cases, ++12 cases). The frequency and grades of antibodies expression are shown in figure above. Abbreviations: anti-nRNP, anti-nuclear ribonucleoprotein antibody; anti-Sm, anti-Smith antibody; anti-SSA, anti-Sjögren's syndrome A antibody; anti-Ro52, anti-52-kDa Ro/SSA antibody; anti-SSB, anti-Sjögren's syndrome B antibody; anti-Scl-70, anti-topoisomerase I; anti-PM/Scl, anti-polymyositis-scleroderma antibody; anti-Jo-1, anti-histidyl-tRNA synthetase antibody; anti-CENP-B, anti-centromere protein B antibodies; anti-PCNA, anti-proliferating cell nuclear antigen antibody; anti-dsDNA, anti-double-stranded DNA antibody; ANuA, Anti-nucleosome antibody; AHA, anti-histone antibody; anti-RPP, anti-ribosomal P protein antibody; AMA-M2, anti-mitochondrial M2 antibody.
